# Comment on ‘Red and processed meat consumption and risk of pancreatic cancer: meta-analysis of prospective studies’

**DOI:** 10.1038/bjc.2012.111

**Published:** 2012-07-17

**Authors:** D Lightsey, National Council Against Health Fraud and Quackwatch

**Affiliations:** 1 Department of Family and Consumer Education, Bakersfield College, Bakersfield, CA, USA; 2 Glinn and Giordano Physical Therapy Inc., Bakersfield, CA, USA

**Sir,**

Larsson and Wolk’s (2012) paper ‘Red and processed meat consumption and risk of pancreatic cancer: meta-analysis of prospective studies’ contains a number of control problems, which makes their conclusions improbable. In the article, the authors state they have identified 11 prospective studies for their meta-analysis, but then admit the inherent problems with these studies as well as their own. Specifically, they state that, ‘our study has some limitations. First, as a meta-analysis of observational studies, we cannot rule out that individual studies may have failed to control for potential confounders, which may introduce bias in an unpredictable direction’. They also declare that ‘only a few studies adjusted for other potential confounders such as body mass index and history of diabetes’. Further, they add that ‘our findings were likely to be affected by imprecise measurement of red and processed meat consumption and potential confounders’. At this point, it would seem to be simple common sense or even prudent to have abandoned the analysis when you know that the data you are trying to extract information from, is itself inherently flawed. ‘This is a meta-analysis of 11 studies, none of which on their own link processed meat-eating with pancreatic cancer. So 11 × 0=zero. All the underlying studies suffer from the usual fatal flaws of weak association epidemiology: here that means, no knowledge of actual meat consumption, confounding risk factors for pancreatic cancer or biological plausibility’ (Milloy, 2012). The failure to make allowances for fruit and vegetable intake, fat mass, and exercise habits are just a few of the confounders absent that may have a major role in pancreatic cancer development. The phrase, ‘garbage in garbage out’ appears to apply to this study. Does combining a number of weak or flawed studies make for a stronger one? What is the point? Who benefits? Certainly not the consumer who has been misled by the headlines associated with this study.

The author’s statement that ‘a positive association between processed meat consumption and risk of pancreatic cancer is biologically plausible’, has not been demonstrated. They do not even cite any direct, long-term case studies using normal exposure levels to support this statement. At best, the association is speculative and weak, certainly not ‘plausible’. The basic principle of toxicology applies here: the dose makes the poison. The amount of nitrites consumers are exposed to as additives in processed meats is irrelevant compared with the amounts from naturally occurring sources, or even the amount that is converted naturally in our saliva from nitrate intake. As an example, fasting saliva contains ∼2 mg l^−1^ of nitrite and after consumption of an amount of nitrate equivalent to 200 g of spinach (about 1 ¼ cup cooked), the nitrite concentration in saliva from its conversion from the nitrate in the spinach may rise as high as 72 mg l^−1^ (Katan, 2009). Additionally, approximately 8% of ingested nitrate can be converted to nitrite (Lundberg *et al*, 1994). The average European ingests nitrate at ∼185 mg per day and the average American ∼100 mg per day (Hord *et al*, 2009). At the 8% conversion rate, this comes to roughly 14.8 and 8 mg per day, respectively. The authors state that by simply ingesting 50 g of processed meat per day, about one serving ‘was associated with a 19% increased risk of pancreatic cancer’. To illustrate what 50 g of processed meat actually brings to the plate, let’s look at 50 g of bacon (roughly two slices), which contains 0.19 mg of nitrite, 50 g of a hot dog (one) would contain 0.025 mg of nitrite and 50 g of ham (∼2 oz) would contain 0.45 mg of nitrite (Hord *et al*, 2009). So what does all this supposed to mean on a practical level, according to this study and the news reports?

It appears that I normally consume roughly 8 mg per day of nitrite from food alone, not counting what I may have consumed from the drinking water. So, according to this study, my added intake of 2 oz of bacon (50 g) with 0.19 mg of nitrite or 2% of my total normal intake is going to raise my risk for pancreatic cancer by 19%. Are you serious? Let’s take another look at this. If increasing my nitrite intake by just 2% causes this much of a potential spike in my pancreatic risk, then what happens when I follow the healthy advice of adding 50 g of spinach (∼1/4 cup cooked) per day to my diet, which naturally contains roughly 370 mg nitrate that in turn would convert into a whopping 29.6 mg of nitrite at 8% conversion rate or 18.5 mg at the lower more conservative conversion rate of 5%? If 0.19 mg of nitrite is associated with the 19% increased cancer rate then what are my chances of survival if I consume spinach? Should I forget the spinach and bring on the ice cream?

The bottom line is that there is no direct evidence of nitrite or nitrates causing human carcinogenicity in humans at normal exposure levels, and the health attributes to these compounds in human biology is expanding. We have understood for decades that those population groups whose diets are low in fruits and vegetables, as well as other poor lifestyle choices, have an increased rate of cancers. In addition, the association of those who are at the highest quartile intake levels of red and processed meats with the associated increased rates of cancer is old information as well as common sense. Many compounds are carcinogenic in animal models in high dosages, but perfectly safe to humans in low dosages. Caffeic acid in coffee is a good example of this, and there are many others.

Lastly, the study relies on epidemiological data (recall data), which I do not believe can determine a cause and effect relationship. Are the media outlets who are reporting such an inference justified? Of course not. But this does not prevent such media headlines associated with this study, such as ‘Study links processed meat with increased risk of pancreatic cancer’ (USA Today, January 13 2012), ‘The hidden risk of processed meats’ (Fox News, January 13 2012), and ‘Two slices of bacon a day increases cancer risk by a fifth, study says’ (Fox News, January 13 2012), from appearing and taking this study out of context. As a health educator as well as a consumer advocate with several consumer groups, I find the ongoing use of weak epidemiological associations as fodder to instill unnecessary fear in consumers unfortunate. Many consumers are now going to be led to believe that the miniscule amount of nitrite used in processed meats, as compared with what is naturally present in many vegetables, and are going to put them at greater risk for pancreatic cancer. In fact, however, the study simply illustrated what we have known for decades: poor lifestyle choices may put consumers at risk for a number of cancers. But, then, we knew that.
